# Dressing of New Born Infants

**Published:** 1866-10

**Authors:** 


### Dressing of New Born Infants.

Dr. W. B. Fletcher, of Indianapolis, Ind., communicates to the *Cincinnati Lancet and Observer*, some remarks on the above subject that are worth reproducing. He says:

If there be one custom of time-honored folly which we have continued to this day in the "lying-in chamber," it is that absurd and cruel system of the first dressing. There is no reason for quoting from the most ancient authors to find absurdity upon this point, when our most recent text-books and lecturers give almost the same directions. But even if they did not, how many physicians ever personally attend to this important point, whereby the comfort of the child and mother are all at stake. In most cases, as soon as the child is born and the cord divided, it is tried, and the baby given to an employed nurse, some wise neighbor or friend. The question of "What will she do with it?" may best be solved by watching her. First she huddles it up in an old shawl or other garment. She is careful to cover its head, as though it were a young puppy she would smother, or rid the world of an infant cat. In a few moments some one brings water, soap, and towels, and also a heap of old linen and a trunk full of new. The good woman now turns to the blazing fire or the hot stove, that the baby may not take cold, and while the youngster implores with yells and cries, she bakes its tender skin on one side, while she dabbles its head, eyes, mouth, and body with a vile solution of frequently very bad soap. After this ceremony has been past, (it matters not whether the child be cleaner than before,) she turns her attention to the cord, upon which she frequently deposits, slyly, some pestiferous saliva. "It's healin'," she says, and now she follows authority. 1st. She cuts or burns a hole in the center of a bit of cloth, through which she draws the cord; 2d. She places a rag upon this; 3. A rag upon that; and 4th. She puts on a "binder." Now it is upon this operation she prides herself if she be a hireling, that is the closeness and compactness with which she can pin the binder round the expanding body of the infant; 5th. She puts on a little garment, called a shirt, which is in fact without body, neck, or sleeves, as far as protection goes; 6th. She puts on the "square," with more pins; 7th. She pins on a "waist" with a long skirt; 8th. Another waist with a longer skirt; 9th. A dress. And now the baby is presentable. The doctor sees it's all right and goes home. He hears not within an hour the stifled screams of compressed lungs, that with every breath are expanding the chest, and the nurse wisely says it appears "colicky," for which it must be drenched with some damned decoction of catnip, sling, brandy, laudnum, water and molasses, etc.

The next visit, the nurse swears its a good child, only a little "colicky," but she can cure that, and away the doctor goes, where he cannot hear the little one cry, and see it dosed for screaming on account of the "cord" having become a half putrid, drying mass, glued and ulcerating to the tender belly.

Dr. Fletcher recommends the following mode of procedure, as an improvement on the above:

"My baby is first quickly washed by oiling the hand and rubbing the parts to which the secretions have adhered, and then with a soft cloth, soft water, and a trace of Castile soap, and frequently with warm water alone, the infant may be cleaned. Then I begin dressing. 1st. A bit of lint or linen, two inches square, is tied closely upon the end of the cord like a cap; 2d. The square, or diaper, of soft and old material, is put on loosely with a diaper-pin; 3d. A fine warm flannel gown, (like a woman's night dress,) with long sleeves, and coming below the feet, is put on, and thus the baby is quickly and comfortably dressed, and placed in its mother's arms, where the temperature of her own body is food and strength for her new-born babe until the milk is secreted.

Let any physician try this plan, and he will meet with opposition from every old lady in the land. 'Why, doctor, its bowels will burst out when it cries, if you don't pin a binder on!' and a number of similar excuses for not being directed by the physician. But the physician will be rewarded by finding the infants more clean, sleeping more, and eating more than when uncomfortably dressed, and I believe less liable to umbilical hernia and ulceration about the cord. I have known children rescued from apparent suffocation by simply unpinning a close binder."—*Medical and Surgical Reporter.*

